# Dynamic expression patterns of ATF3 and p53 in the hippocampus of a pentylenetetrazole-induced kindling model

**DOI:** 10.3892/mmr.2014.2256

**Published:** 2014-05-21

**Authors:** DONG-XUE DING, FA-FA TIAN, JIA-LING GUO, KAI LI, JING-XUAN HE, MING-YU SONG, LI LI, XIA HUANG

**Affiliations:** 1 Department of Neurology, Xiangya Hospital, Central South University, Changsha, Hunan 410008, P.R. China; 2 Science Research Center, Xiangya Hospital, Changsha, Hunan 410008, P.R. China; 3 Department of Neurology, Wangwang Hospital, Changsha, Hunan 410016, P.R. China

**Keywords:** epilepsy, activating transcription factor 3, p53, axon growth, MFS

## Abstract

Epilepsy is a common and often deleterious neurological condition. Emerging evidence has demonstrated the roles of innate immunity and the associated inflammatory processes in epilepsy. In a previous study, we found that Toll-like receptors (TLRs) are upregulated and promote mossy fiber sprouting (MFS) in an epileptic model. As downstream effectors of TLRs, the activating transcription factor 3 (ATF3) and p53 proteins were shown to be involved in neurite outgrowth. In the present study, we hypothesized that ATF3 and p53 participate in the process of epilepsy and can affect MFS. To investigate this hypothesis, we examined the expression of ATF3 and p53 in hippocampal tissues of rats kindled by pentylenetetrazole (PTZ) using immunofluorescence, immunohistochemistry and western blotting. MFS was evaluated by Timm staining in the hippocampus. Results from these experiments revealed that expression of ATF3 and p53 is significantly higher (p<0.05) in the CA3 area of the hippocampus in the PTZ-treated group compared to the control group. ATF3 expression gradually increased from 3 days to 4 weeks, peaked at 4 weeks and decreased slightly at 6 weeks in the PTZ group, while the expression of p53 was maintained at similar levels at different time-points following PTZ treatment. No obvious difference in the expression of these proteins was observed between the PTZ and the control group in the dentate gyrus (DG) area (p>0.05). The degree of MFS in the PTZ group peaked at 4 weeks and was maintained at a high level until 6 weeks post-PTZ treatment. In conclusion, ATF3 and p53 may be involved in the occurrence of seizure and play critical roles in MFS in the PTZ kindling model.

## Introduction

Epilepsy, a disorder of recurrent seizures, is a common and often deleterious neurological condition. It has considerable impact on the patients’ quality of life and greatly increases the risk of injury, socioeconomic disadvantage, and even mortality (1). It can also interfere with memory, cognitive function and education opportunities, and it may cause endocrine dysfunction (2). Despite the development and availability of >22 anti-epileptic drugs (AEDs), most of which have been identified based on large-scale, randomized, double-blind clinical trails, it is estimated that 25–40% of patients diagnosed with epilepsy are resistant to drug therapy and continue to have seizures (3,4). Thus, exploring the molecular mechanisms underlying epilepsy may allow to identify novel treatment methods.

The roles of the inflammatory system in the occurrence of seizure are currently heavily investigated. Louboutin *et al* (5) showed that the C-C chemokine receptor type 5 is involved in neuronal injury caused by kainic acid (KA) in animal models. Our previous study reported that Toll-like receptors (TLRs) contribute to the development of epilepsy (6). The majority of TLRs recruit the MyD88-IRAK-TRAF6 pathway, culminating in the activation of nuclear factor-κB, which drives the transcription of genes encoding pro-inflammatory factors, such as interleukin (IL)-6, IL-12, and tumor necrosis factor-α (7). Earlier studies by Whitmore *et al* (8) and Gilchrist *et al* (9) demonstrated that the activating transcription factor 3 (ATF3) is induced by TLR signaling in primary mouse macrophages and human dendritic cells. ATF3 modulated the transcription of IL-6, IL-12b, and IL-12p40, which highlights its key regulatory roles in TLR signaling. Recent studies have further reported that *TLR* gene expression closely interacts with that of *p53* (10,11). The hypothesis that ATF3 can modulate the activity of p53 was based on evidence supporting the interaction between these two proteins (12).

A previous study in an epileptic rat model suggested that aberrant mossy fiber sprouting (MFS) may contribute to spontaneous seizures (13). A recent study by our group showed that TLRs modulate neurite outgrowth in the hippocampus of pentylenetetrazole (PTZ)-kindled rats (6). In addition, other studies found that overexpression of ATF3 plays a crucial role in promoting neurite outgrowth in the peripheral nervous system, both *in vitro* and *in vivo* (14–16). The tumor protein p53 has been also shown to promote neurite growth: overexpression of a dominant negative form of p53 in primary cortical neurons led to growth cone collapse and a decrease in neurite outgrowth (17,18). In this context, we hypothesized that ATF3 and p53 may be involved in MFS during epileptogenesis. To verify this hypothesis, we established a kindling model of epilepsy via intraperitoneal injection of PTZ in rats, and analyzed the expression level of the ATF3 and p53 proteins.

## Materials and methods

### Animals and drug treatment

Rats were treated following the Guidelines for the Care and Use of Laboratory Animals, published by the National Institutes of Health (NIH; Bethesda, MD, USA). All protocols were approved by the Animal Ethics Committee of the Central South University in China. A total of 180 adult male Sprague-Dawley rats (6–8 weeks of age, 180–220 g) were purchased from the Animal Experimental Center of the Central South University (Changsha, China). They were housed in quiet rooms with a 12–12 h light-dark cycle (light from 07:00 a.m. to 19:00 p.m.) and a 22–24°C temperature, and were given standard laboratory food and tap water *ad libitum*. The rats were randomly divided into the control and PTZ groups, each containing 5 subgroups of 18 rats each. There was no statistically significant difference in weight and age between rats of the two groups. Rats of the PTZ group received an intraperitoneal injection of 30 mg/kg PTZ (Sigma-Aldrich, St. Louis, MO, USA) every day until they were kindled or sacrificed, while those of the control group were injected with an equal dose of normal saline. The rat behavior was recorded by a video camera (Sony Corp., Tokyo, Japan). Following PTZ injection, the rats were monitored for a minimum of 2 h to assess the severity and duration of the seizures. Rats were considered kindled when seizure attacks (Racine’s scale score ≥3) occurred after each PTZ injection for 5 consecutive days. At 3 days and 1, 2, 4 and 6 weeks after the first injection, the rats were sacrificed and perfused.

### Immunohistochemistry

At different time-points, the rats were deeply anesthetized with 10% chloral hydrate and perfused intracardially with 300 ml of normal saline and 400 ml of 4% paraformaldehyde in 0.1 M phosphate buffer at 4°C. The brains were removed and placed in 4% paraformaldehyde overnight, then transferred into 0.1 M phosphate buffer containing 20–30% sucrose. Subsequently, serial, 20-μm-thick sections were performed for immunohistochemistry and immunofluorescence analysis. The sections were subjected to conventional rewarming and heat-induced antigen retrieval in 10 mM sodium citrate buffer (0.01 mol/l; Sinopharm Chemical Reagent Co., Ltd., Shanghai, China) at boiling temperature for 24 min; cool sodium citrate buffer was added every 6 min. Peroxidase and lipids were eliminated by the admixture of 1% hydrogen and methanol at 4°C for 30 min. After rinsing in 0.01 M phosphate-buffered saline (PBS), the sections were blocked using 5% goat serum at room temperature for 2 h and incubated overnight at 4°C with the rabbit anti-rat monoclonal antibodies anti-ATF3 and −p53 (1:50; Santa Cruz Biotechnology, Inc., Santa Cruz, CA, USA). After rinsing in 0.01 M PBS, the sections were incubated with a biotinylated goat anti-rabbit secondary antibody (Zhongshan Golden Bridge Biotechnology Co., Ltd., Beijing, China) at room temperature for 60 min. To visualize peroxidase labeling, the sections were stained with diaminobenzidine (Boster Biological Technology Ltd., Wuhan, China), dehydrated and mounted. The sections were observed under a fluorescence microscope (Leica DM5000B microscope; Leica Camera Co., Solms, Germany). Images were processed with a Leica DM5000 B image analysis system (Leica Microsystems, Glattbrugg, Switzerland).

### Immunofluorescence

Rewarming and antigen recovery of brain tissue sections were performed as described above. Each section was permeabilized with 1% Triton X-100 in Tris-buffered saline with Tween-20. After blocking with 10% goat serum (Zhongshan Golden Bridge Biotechnology Co., Ltd.) at room temperature for 2 h, the samples were incubated at 4°C overnight with anti-ATF3 or −p53 at 1:12.5 dilution. After rinsing in 0.01 M PBS, the sections were incubated in the dark for 1 h at room temperature with Alexa Fluor 555-conjugated goat anti-rabbit IgG (1:1,000; Invitrogen, Carlsbad, CA, USA). The fluorescence intensity was measured on a Leica DM5000 B system.

### Western blotting

The entire hippocampi, including both CA3 and dentate gyrus (DG) areas, were used for western blot analysis. Rats in the control and the PTZ groups were deeply anesthetized with chloral hydrate (350 mg/kg), and cervical dislocation was performed at different time-points. Tissues were snap-frozen in liquid nitrogen, and protein samples were extracted directly from the hippocampi by homogenization in admixture of 1 mM phenylmethyl sulfonyfluoride (PMSF) and RIPA buffer (Beyotime Institute of Biotechnology, Shanghai, China). Following heating at 100°C for 10 min in 5X SDS-PAGE loading buffer, (Beijing Cowin Biotech Co., Ltd., Beijing, China), equal amounts of denatured protein were separated by 10% sodium dodecyl sulfate (SDS)-polyacrylamide gel electrophoresis, and the protein bands were electrotransferred onto polyvinylidene fluoride (PVDF) membranes (Pall Corp., Port Washington, NY, USA) and stained with the appropriate antibody (anti-ATF3, 1:1,000; anti-p53, 1:1,500). Immunostaining with the 3-phosphate dehydrogenase (GAPDH) antibody (1:2,000; Sigma-Aldrich) was used to normalize the expression data. The immunoreactive bands were visualized by enhanced chemiluminescence using Image Lab™ software with the gel imaging analysis system (Bio-Rad, Hercules, CA, USA).

### Timm staining

At different time-points, the rats were deeply anesthetized with 10% chloral hydrate (Laboratory of The Second Xiangya Hospital, Central South University, Changsha, China) and perfused intracardially with 300 ml of normal saline, followed by addition of 200 ml of 0.1 M phosphate buffer (pH, 7.2–7.6; Sinopharm Chemical Reagent Co., Ltd.) containing 0.4% sodium sulfide (Shanghai Aibi Chemistry Preparation Co., Ltd., Shanghai, China) and 400 ml of 4% paraformaldehyde (Tianjin Chemical Reagent Co., Ltd., Tianjin, China), at 4°C. The brains were removed, fixed in 4% paraformaldehyde for 24 h, transferred to 0.1 M phosphate buffer containing 30% sucrose (Sinopharm Chemical Reagent Co., Ltd.), and cut into 30-μm coronal sections. The sections were stained in the dark for 90 min in a solution containing 60 ml of 50% arabic gum (Sinopharm Chemical Reagent Co., Ltd.), 10 ml of 2 M citrate buffer (Sinopharm Chemical Reagent Co., Ltd.), 30 ml of 0.5 M hydroquinone (Shanghai Aibi Chemistry Preparation Co., Ltd.) and 0.5 ml of 17% silver nitrate (Sinopharm Chemical Reagent Co., Ltd.). The glass slides were washed in de-ionized water and counterstained by Nissl solution (Beyotime Institute of Biotechnology, China) for 5 min. Subsequently, the glass slides were dehydrated with gradient ethanol between 50 and 100%. They were made transparent by xylene and mounted with permount mounting medium (Sinopharm Chemical Reagent Co., Ltd.). Mossy fiber sprouting was evaluated by rating the distribution of supragranular Timm granules (TG) at a standard location in the dorsal and the ventral hippocampus. Timm scoring scale ranged between 0 and 5 according to the following criteria: 0, no TG in the supragranular region; 1, sparse TG in the supragranular regions in a patchy distribution; 2, several TG in a continuous distribution; 3, prominent TG on a continuous distribution with occasional patches of confluent TG; 4, prominent TG forming a confluent dense laminar band and 5, a confluent dense laminar band of TG that further extends into the inner molecular layer.

### Statistical analysis

Statistical analysis was performed with the GraphPad Prism 5 software (GraphPad Software, Inc., La Jolla, CA, USA), and the data were expressed as the mean ± standard deviation (SD). Differences among multiple groups were assessed by a one-way analysis of variance (ANOVA), and differences between 2 groups were evaluated using the independent samples t-test. Differences with p<0.05 were considered significant.

## Result

### Behavior of PTZ-treated rats

With the exception of 5 rats in the PTZ group that died as a result of status epilepticus or generalized clonic-tonic seizures at 1 or 2 weeks, the remaining rats in this group developed seizure activity of stage 3, 4 or 5 after continuous PTZ injection for 18–22 days. The PTZ-induced seizure activity generally occurred 5–10 min after the PTZ injection, and had a duration of 5–30 min. Spontaneous recurrent seizures of grade 2–3 were detected in kindled rats as early as 23 days after the first injection. No epileptiform activity was observed in the control groups.

### The severity of MFS in the CA3 region is associated with the evolution of seizure behavior

**Figure 1:**
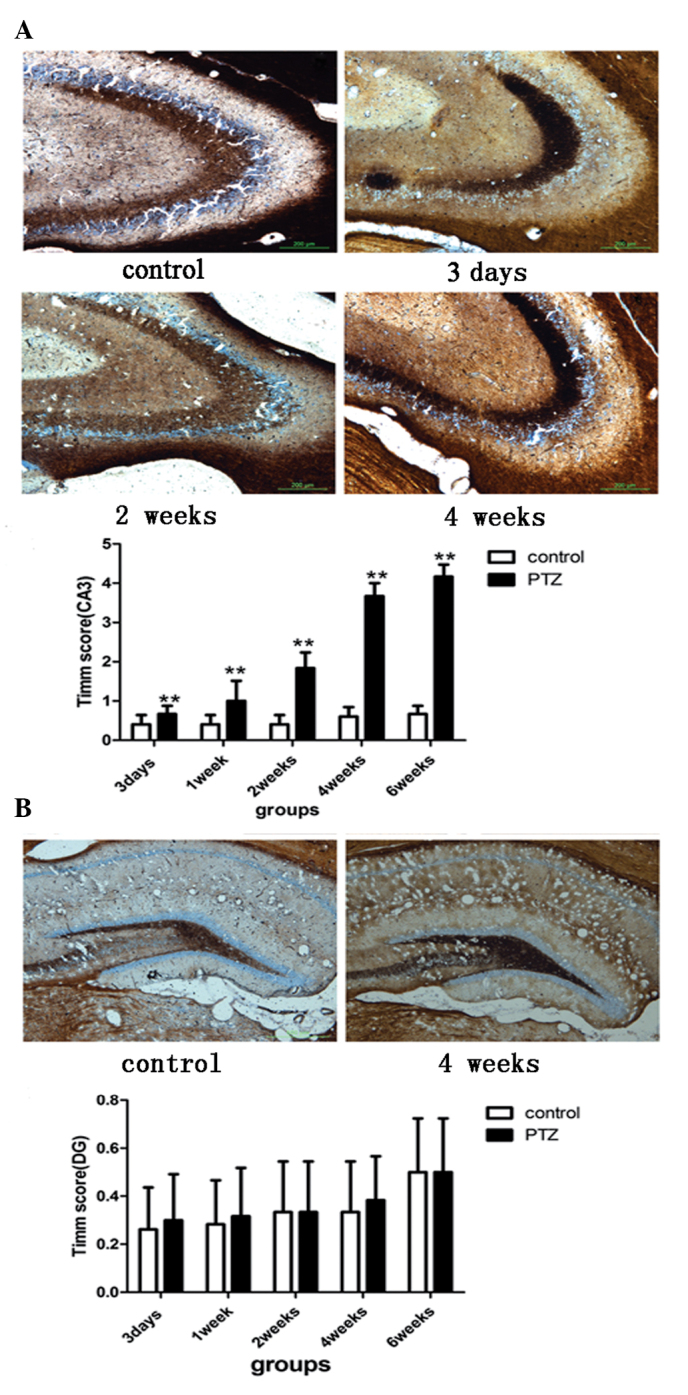
Timm staining in the (A) CA3 and (B) dental gyrus (DG) inner molecular layer areas (IML) of the control and the pentylenetetrazole (PTZ) groups. Upper panels show Timm granules from the control and the PTZ groups (different time-points following treatment), as observed under a fluorescence microscope (magnification, ×100). Scale bars (green lines), 200 μm. Lower panels show quantified intensities of Timm granules (Timm scores), calculated in 28 PTZ and 30 control rats. ^**^P<0.05. Timm scores gradually increase following PTZ injection in the CA3 area. No significant increase in Timm scores, indicative of fiber sprouting (MFS), is observed in the CA3 area of the control group, and in the IML of either the control or the PTZ group.

The Timm scores in the CA3 area of the PTZ group were significantly different from those of the control group in all time-points (p<0.05; Fig. 1A), and were prominently increased at 2, 4 and 6 weeks after the first injection. The degree of MFS in the CA3 area of the PTZ group, indicated by the corresponding Timm scores, was consistent with the grade of seizures. At 3 days and 1 week post-PTZ treatment, most of the rats in the PTZ group did not show epileptic seizures. At 2 weeks, the pathology of most of the rats did not change, with only few rats showing head myoclonus (n=3) or forelimb clonus (n=5). At 4 and 6 weeks, most of the rats in the PTZ group were kindled, and MFS reached its highest degree as compared to other time-points. In the DG area, the Timm scores ranged between 0 and 1 for all rats. There was no significant difference in MFS between the control and the PTZ groups in this area (p>0.05; Fig. 1B).

### The expression of ATF3 and p53 in the CA3 areas is significantly increased during progression of PTZ-induced kindling

**Figure 2:**
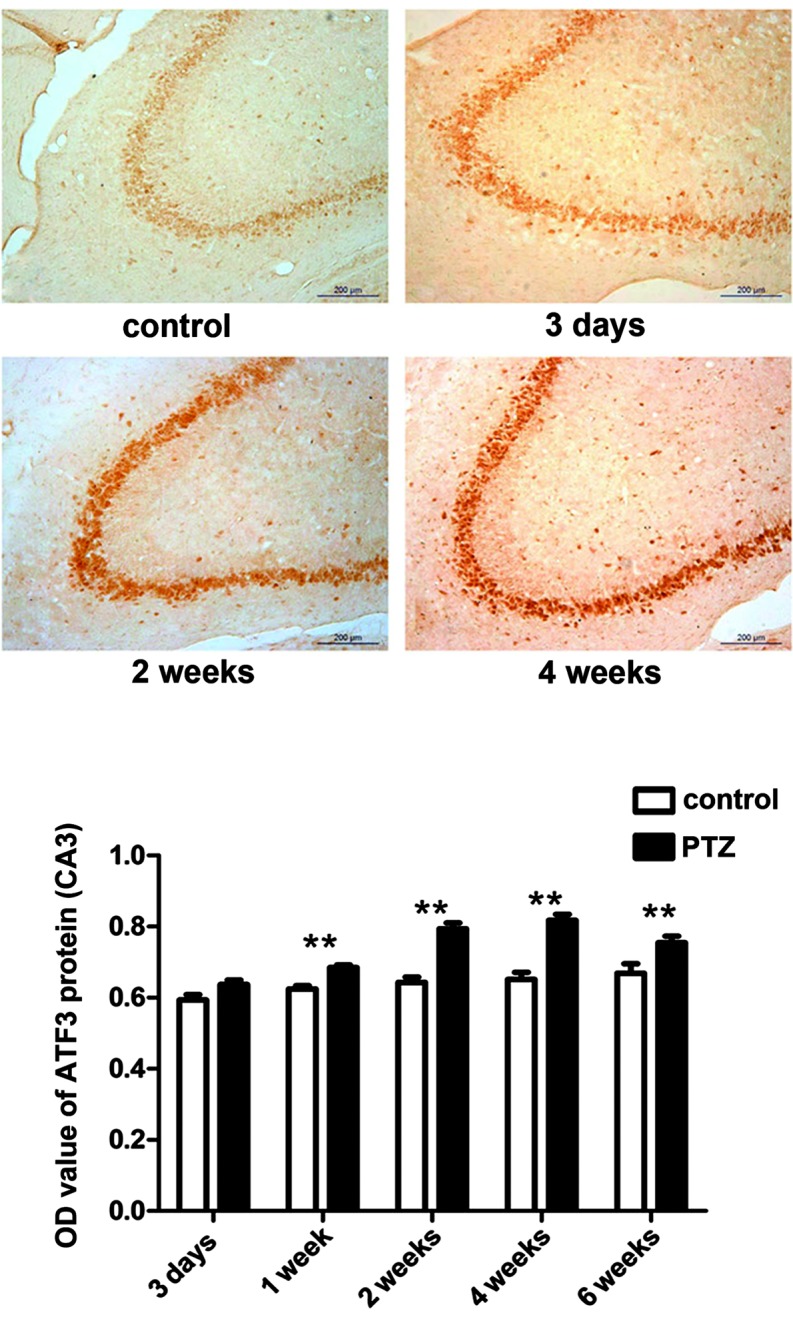
The expression of activating transcription factor 3 (ATF3) in the CA3 area of the hippocampus, as assessed by immunohistochemical staining in the control and the PTZ groups (different time-points post-treatment). The upper panel shows AT3 staining as observed under a fluorescence microscope (magnification, ×100; scale bars, 200 μm). The lower panel shows quantified intensities from 29 PTZ rats and 30 control rats. Compared to the control group, ATF3 expression is significantly increased as early as 1 week post-PTZ injection, reaches a peak at 4 weeks, and shows a slight decline at 6 weeks. ^**^P<0.05.

**Figure 3:**
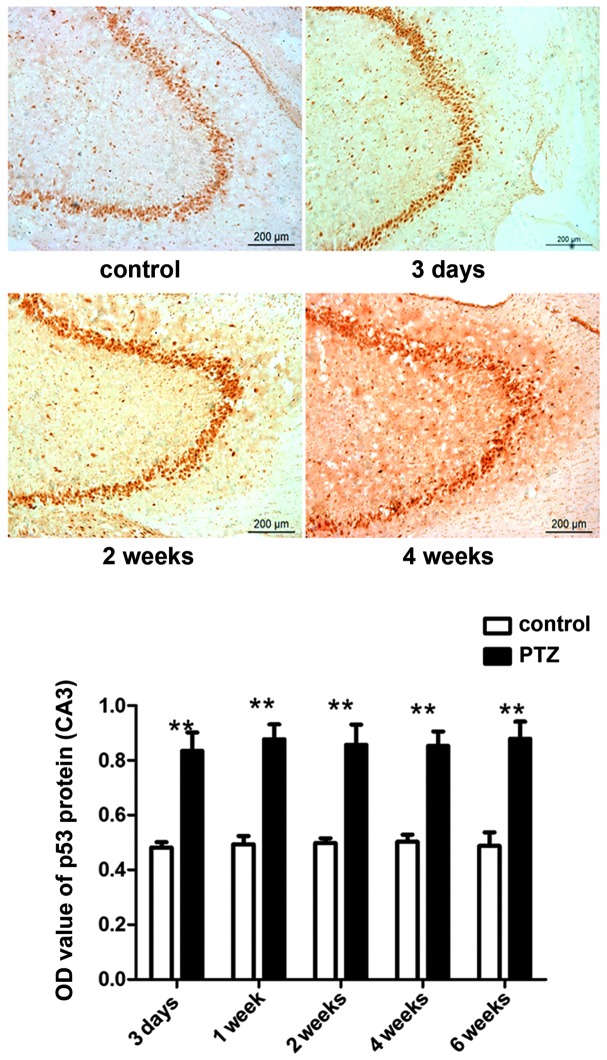
The expression of p53 in the CA3 area of the hippocampus, as assessed by immunohistochemical staining in the control and the PTZ groups (different time-points post-treatment). The upper panel shows p53 staining as observed under a fluorescence microscope (magnification, ×100; scale bars, 200 μm). The lower panel shows quantified intensities from 29 PTZ and 30 control rats. p53 shows higher expression in the PTZ group compared to the control, but was expressed at similar levels (P>0.05) from 3 days to 6 weeks.

**Figure 4:**
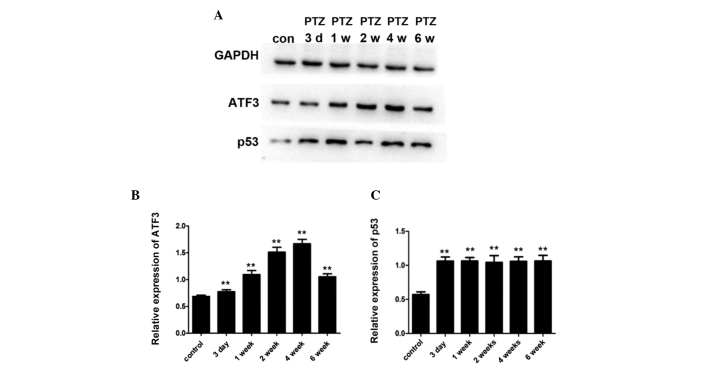
The expression of activating transcription factor 3 (ATF3) and p53 in the hippocampus. (A) Western blot of samples from 28 PTZ and 30 control (con) rats. The 3-phosphate dehydrogenase (GAPDH) was used as the loading control. D, days; w, weeks. Quantification of the western blot results showed that (B) ATF3 expression is increased at all time-points in the pentylenetetrazole (PTZ) group compared to the control; it gradually increases from 3 days to 4 weeks, and decreases from 4 to 6 weeks; and that (C) the expression of p53 is increased in the PTZ compared to the control group.

**Figure 5:**
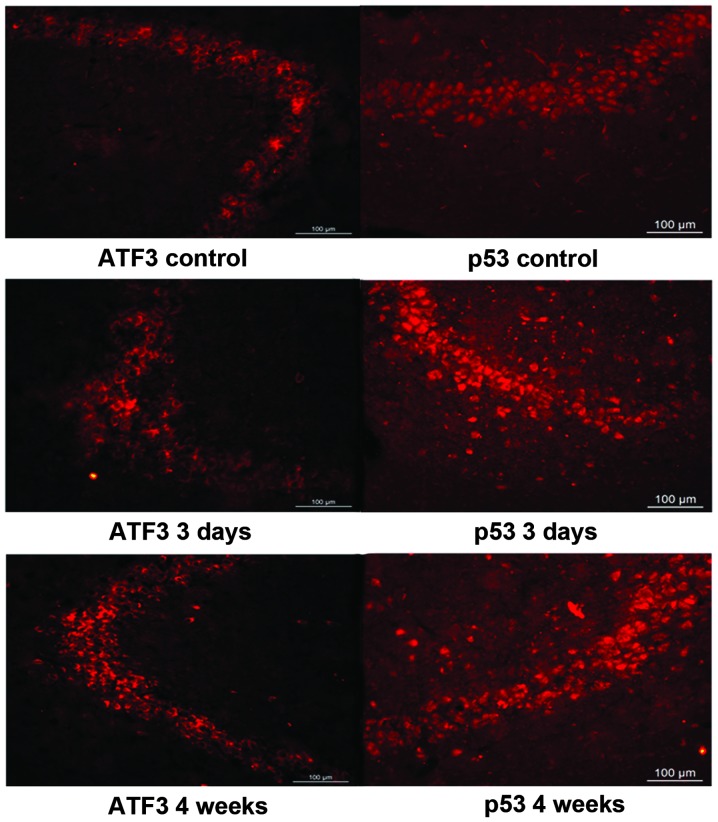
Immunofluorescence staining of activating transcription factor 3 (ATF3) and p53 in the CA3 area of the hippocampus, as observed under a fluorescence microscope (magnification, ×200). ATF3 mainly accumulates in the cytoplasm of the neurons, while p53 mostly locates in the cell nuclei. 29 PTZ and 30 control rats were assessed by immunofluorescence. Scale bars, 100 μm.

The expression of the ATF3 and p53 proteins was mainly observed in the pyramidal cells in the CA3 region and in hilar neurons in the DG within the hippocampus. Compared to the control group, expression of ATF3 and p53 in the CA3 area of the PTZ group was significantly increased (P<0.05). Immunohistochemistry analysis showed that the expression of ATF3 in the PTZ group gradually increased from 3 days to 4 weeks, peaked at 4 weeks, and slightly decreased at 6 weeks (Figs. 2 and 4). The expression of p53 was also higher in the PTZ group compared to the control group, but no significant difference (P>0.05) was observed between 3 days and 6 weeks following PTZ treatment (Figs. 3 and 4). No obvious differences in the expression of the two proteins were observed in the DG area between the PTZ and the control group (data not shown). Figs. 4 and 5 show western blot and immunofluorescence analysis results, confirming the above-described patterns of ATF3 and p53 expression. ATF3 mainly accumulated in the cytoplasm of the neurons in the CA3 area of the hippocampus, while p53 mostly localized in the cell nuclei in the CA3 area of the hippocampus.

## Discussion

Increasing evidence has highlighted the roles of immunity and inflammatory processes in epilepsy (19–23). Our previous study demonstrated that TLR signaling contributes to the occurrence of epilepsy, and that suppressors of cytokine signaling may act as negative regulators of TLR signaling (6). In the current study, we studied the dynamic changes in the expression of the TLR downstream effectors ATF3 and p53 in the hippocampus of a PTZ-induced kindling rat model.

The expression of both ATF3 and p53 proteins was increased in the PTZ compared to the control group, indicating that these proteins may participate in the occurrence of epilepsy. ATF3 is encoded by an early-response gene, the expression of which is induced in cells exposed to a variety of stress stimuli (24,25). It was previously proposed that there is a regulatory feedback regulation loop between p53 and ATF3 (26). In the nervous system, ATF3 appears to contribute to the regenerative response (27,28), and p53 was found to be involved in neurite outgrowth and nerve regeneration (18). In our study, the expression of ATF3 in the CA3 area of the hippocampus gradually increased and then slightly decreased during the studied period. The ATF3 expression profile and the degree of MFS in the CA3 area were concordant over time, as well as their localization. Our results indicated that ATF3 may modulate neurite outgrowth and affect neurogenesis in the hippocampus during the process of kindling. These results are however not in agreement with those reported by Francis *et al* (29), which may be probably attributed to the different type of convulsants used. Compared to KA used in the study by Francis *et al*, PTZ is a relatively mild convulsant that leads to a more mild cell death. Therefore, the main function of ATF3 may differ in different experimental models. The expression of p53 in the PTZ group was not in complete accordance with the degree of MFS; it increased 3 days post-PTZ injection and was maintained at similar levels after this time-point. As Sakhi *et al* (30) reported, p53 acts as a marker of irreversible neuronal injury. In our study, p53 was upregulated at the early stage of the kindling process. This indicates that p53-induced neuronal death may occursonly during the early stages of epileptogenesis, and that p53 may be an important factor in maintaining neurite outgrowth.

In addition, it has been reported that p53 is involved in ATF3-mediated injury response, potentially via ATF3-mediated regulation of the p53 stability, by interference upon p53 ubiquitination (31). In a study by Yan *et al* (32), ATF3 was found to directly bind to p53 and repress the p53-dependent transactivation of the type IV collagenase gene (*MMP-2*) promoter. The expression patterns of the ATF3 and p53 proteins were highly similar during the kindling process in our experiments. We hypothesize that p53 may be one of the perpetuating factors in the development of MFS, since its expression increased before manifestation of MFS, and was maintained at similar levels afterwards. Following the injection of PTZ, p53 expression was maintained at high levels, probably in order to promote the development of MFS. Considering the consistency in ATF3 expression and the changes in the degree of MFS, ATF3 may be more important in promoting the development of MFS. Whether there is an interaction between ATF3 and p53 needs to be further investigated.

In summary, our results demonstrated an increase in the levels of ATF3 and p53 in the PTZ groups compared to the control groups in the rat hippocampus. This study indicated that ATF3 may play a role in aberrant MFS in a p53-dependent manner during the early stage of epileptogenesis. We did not knockout or overexpress the rat ATF3 and p53 genes or proteins in this study; these experiments may provide additional evidence to explain our results and to elucidate the functions of the two proteins. Further studies are needed to elucidate their roles in the neurons, both in rat models and epileptic patients. The results reported herein provide important perspectives for future studies on ATF3 and p53, aiming to identify more effective treatments for epilepsy.
